# Spartin is a Lipid Transfer Protein That Facilitates Lipid Droplet Turnover

**DOI:** 10.1177/25152564241255782

**Published:** 2024-05-26

**Authors:** Yaoyang Zhong, Tim P. Levine

**Affiliations:** 1 UCL Institute of Ophthalmology, University College London, London, UK

**Keywords:** lysophagy, autophagy, lipophagy, spastin, membrane contact sites

## Abstract

One means by which cells reutilize neutral lipids stored in lipid droplets is to degrade them by autophagy. This process involves spartin, mutations of which cause the rare inherited disorder Troyer syndrome (or spastic paraplegia-20, SPG20). A recently published paper from the team led by Karin Reinsich (Yale) suggests that the molecular function of spartin and its unique highly conserved “senescence” domain is as a lipid transfer protein. Spartin binds to and transfers all lipid species found in lipid droplets, from phospholipids to triglycerides and sterol esters. This lipid transfer activity correlates with spartin's ability to sustain lipid droplet turnover. The senescence domain poses an intriguing question around the wide range of its cargoes, but intriguingly it has yet to yield up its secrets because attempts at crystallization failed and AlphaFold's prediction is unconvincing.

Lipid droplets (LDs) are reservoirs of neutral fat enveloped by a phospholipid monolayer that play key roles in energy storage and lipid metabolism (Olzmann and Carvalho, 2019). An important route of LD metabolism and turnover is by autophagy, a pathway called macro-lipophagy, to distinguish it from micro-lipophagy by which LDs directly enter the lysosome/vacuole without going through an autophagosomal stage. In mammalian cells, but not yeast, both macro- and micro-lipophagy involve LD fragmentation, with the fragments rather than the whole LD being engulfed by lysosomes or autophagosomes (Schulze et al., 2020). Protein-mediated mechanisms for lipid transfer to and from LDs are particularly located at sites of contact between LDs and other organelles (Henne, 2023). For example, seipin at the ER–LD interface may be involved in lipid ester import into LD (Salo et al., 2016), ORP5 regulates LD phosphatidylserine (PS) and phosphatidylinositol-4-phosphate at ER–LD contact sites (Du et al., 2020) and mitoguardin-2 transports lipids between mitochondria and LDs (Hong et al., 2022). Based on the finding that spartin localizes to an LD contact, here with an LC3-positive compartment, therefore either an autophagosome or a lysosome, and that it has a domain of unknown function, the Reinisch team proposed that spartin might also be a lipid transfer protein. The domain structure of spartin comprises three domains and two motifs (Figure 1A). The domains, from N- to C-terminus are: a microtubule interacting and transport (MIT) domain, a pleckstrin homology domain (PH, a region mapped as binding ubiquitin), and a senescence domain, which is ∼210 residues long with no known function, named for plant phenotype associated with deletion of the domain (Ciccarelli et al., 2003).

**Figure 1.**
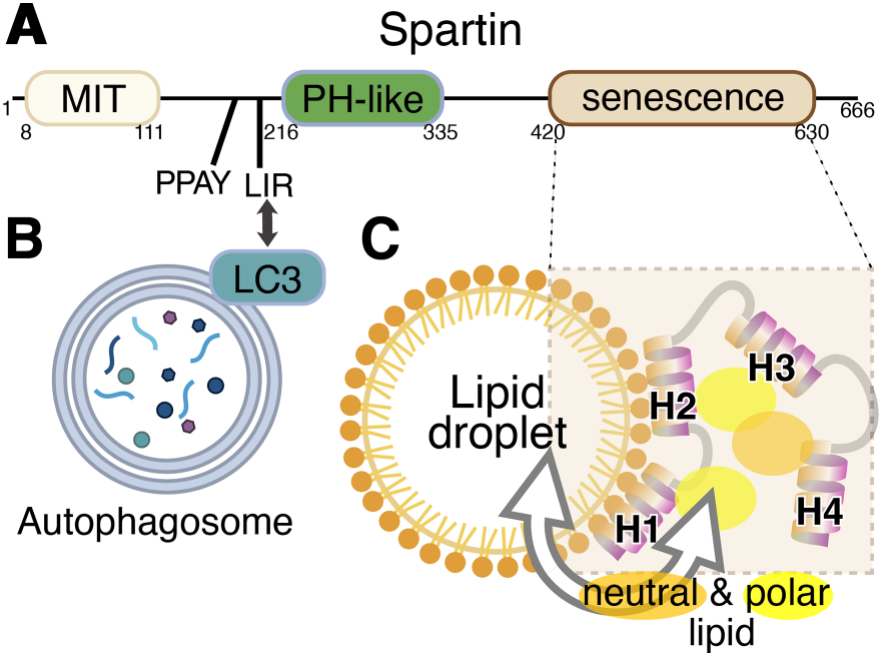
Domain diagram of Spartin protein and model of its interaction with autophagosomes and lipid droplets. (A) Three domains and two motifs in human spartin are shown with their boundaries. (B) The N-terminal LC3A/C-interacting region (LIR) motif targets LC3 on autophagosomes. (C) The C-terminal senescence domain, which interacts with the lipid monolayer of lipid droplets, consists of four amphipathic helices (H1–4) that are capable of solubilizing both polar and neutral lipids. While both pairs of helices H1/2 and H3/4 are required for lipid transfer by spartin, only H1/2 affects its localization to LDs.

Spartin has long been known as a component of LDs (Eastman et al., 2009), and more recently has been shown to interact with the core autophagic machinery to deliver LDs to lysosomes (Chung et al., 2023). Organelle bridging arises from the combination of an LC3A-interacting region (LIR) motif that targets LC3-positive compartments and the senescence domain that binds LDs (Figure 1). LD localization depends on the senescence domain's four amphipathic helices (AHs), which were proposed to integrate into packing defects in the LD phospholipid monolayer (Chung et al., 2023).

In the new study, the senescence domain was found to have a role beyond LD targeting: unexpectedly it can bind and transfer lipids between membrane compartments (Wan et al., 2024). The first hint that it can transfer lipids came from showing that full-length spartin purified *in vitro* can solubilize glycerophospholipids (various headgroups) at a protein:lipid ratio of 1:1. Spartin in cell lysates was then found to contain not only phospholipids (with a bias against negatively charged species) but also neutral lipids, both sterol esters and triacylglycerols, the latter making up the majority of bound species (∼65%). Spartin was also shown to transfer the same range of phospholipids as it binds (for example, phosphatidylcholine but not PS). Importantly, the authors showed that lipid binding/transfer activities all reside in the senescence domain.

Further progress in detailed understanding came through mapping the critical elements among the four AHs that make up the senescence domain. It was shown that deletion of two of the AHs, either the N-terminal pair (H1/2) or the C-terminal pair (H3/4), prevented lipid transfer. Also, recreating a disease-causing mutation that puts a break in H1 (alanine → proline) prevented lipid transfer (Bizzari et al., 2017). For the simpler step of LD association, activity required H1/2 but not H3/4. The senescence domain has no known homologs, even remote ones, so for greater structural insight the authors turned to predicting structure with AlphaFold (Jumper et al., 2021). However, here they realized that the algorithm may not be informative. The four AHs were predicted to have different relationships to each other depending on whether the domain was from human, worm, or fungus, which indicates that the tool cannot produce a single reliable model (Azzaz et al., 2022). In addition, the standard structural crystallography approach also failed – no crystals were obtainable.

To make some progress beyond these obstacles, spartin complexes with endogenous lipids purified from HEK293 cells were analyzed by hydrogen/deuterium exchange mass spectrometry (HDX-MS), which evaluates secondary structure dynamics (Wan et al., 2024). This showed that the A→P mutation that impaired lipid transport mutant has significantly altered hydrogen–deuterium exchange rates in H1 compared to wild-type protein, suggesting that the senescence domain's function in lipid transfer requires a defined tertiary structure. In a final set of experiments, the authors demonstrated that spartin's senescence domain has a second *in vivo* function beyond tethering. As expected, spartin deletion increased LD quantity, with reduced turnover and reduced LD–autophagosome contact. Rescuing this phenotype with plasmid-borne spartin variants showed that H3/H4 were required for spartin to rescue LD turnover. Since the same helices are also required for lipid transfer in vitro, it is proposed that the second function is one and the same as lipid transfer.

Although many gaps remain, the authors hypothesize that spartin might transfer glycerophospholipids paradoxically away from the autophagosome to LDs, providing more phospholipids for LD surface monolayers, so LDs can break down into smaller droplets (requiring a higher surface-to-volume ratio). Another possibility is that spartin transfers a combination of neutral lipids and phospholipids from LDs to autophagosomes.

Among the outstanding questions are whether spartin's lipid transfer activity is required for a newly described role of lysophagy, where it marks damaged lysosomes for destruction rather than repair (Gahlot et al., 2024). More mechanistically, does spartin bind lipid (be it polar or neutral) at a 1:1 ratio or alternately is a single polar lipid accompanied by additional neutral lipid, allowing for a protein:total lipid ratio of 1:3. Finally, the direction of lipid transfer by spartin and its cargoes is unknown. Answering these questions could be made possible by obtaining more structural Information, which is surely a worthwhile future goal, and would lead to an understanding of all the cellular functions of senescence domains.
